# Regioselective semi-synthesis of 6-isomers of 5,8-*O*-dimethyl ether of shikonin derivatives via an ‘intramolecular ring-closing/ring-opening’ strategy as potent anticancer agents

**DOI:** 10.1186/s13065-017-0306-0

**Published:** 2017-08-02

**Authors:** Li Zhou, Xu Zhang, Wen Zhou

**Affiliations:** 1 College of Science, Hunan Agricultural University, Furong, Changsha, 410128 Hunan Province China; 2 College of Forestry and Landscape Architecture, South China Agricultural University, 483, Wushan Rd, Guangzhou, 510642 Guangdong Province China; 3 School of Chinese Meteria Medica, Guangzhou University of Chinese Medicine, E. 232, University Town, Waihuan Rd, Panyu, Guangzhou, 510006 Guangdong Province China

**Keywords:** 6-isomer of 5,8-*O*-dimethyl ether of shikonin, Ring-closing/ring-opening strategy, Bulky substituent, Semi-synthesis, Shikonin, Anticancer scaffold

## Abstract

Synthesis of 6-isomer of 5,8-*O*-dimethyl ether of shikonin (**13**), a promising anticancer scaffold, always remains a huge challenge. Herein a key intermediate for **13**, 2-(1-hydroxyl-4-methyl-3-pentenyl)-1,4,5,8-tetramethoxynaphthalene (**10**), was obtained on the large-scale synthesis. A ring-closing/ring-opening strategy was applied to avoid the undesired reactivity posed by the side chain and racemization of the chiral centre. Incorporation of bulky substituent 4-((*tert*butoxycarbonyl)amino)phenyl to hydroxyl group in the side chain redistributed electron density of naphthalene core (**10**), overwhelmingly favoring the generation of **13** when oxidized by cerium(IV) ammonium nitrate followed by hydrolysis. As a result, three 6-isomers (**14a**–**14c**) with very potent antitumor activity were easily synthesized. This study opened an novel avenue to selectively prepare 6-isomers of 5,8-dimethoxy1-1,4-naphthaquinones, bearing the synthetically challenging side chain such as 2-hydroxyl-5-methylpentenyl group.

**Electronic supplementary material:**

The online version of this article (doi:10.1186/s13065-017-0306-0) contains supplementary material, which is available to authorized users.

**Graphical abstract:**
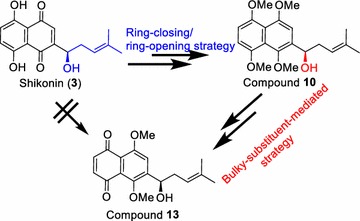
A novel semi-synthesis of 6-isomer of 5,8-*O*-dimethyl ether of shikonin (**13**) was developed in a threestep rounte using a ring-closing/ring-opening strategy and a bulky substituent-mediated oxidative demethylation one

## Background

**Fig. 1:**
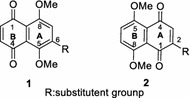
Structures of C-6 or C-2 substituted 5,8-dimethoxyl-1,4-naphthaquinone derivatives

The medical application of *Lithospermum erythrorhizon* extract as an effective therapy for inflammation [1], infectious diseases [2], cancer [2] and atherosclerosis [2, 3] has been known very well for centuries. Its active ingredients, shikonin and its derivatives, have been extensively explored using various semi-synthetic or total-synthetic methodologies. Compounds with different substituents, such as hydroxyalkyl [4], acyl [5], or hydroxyliminoalkyl [6], on C-6 (6-isomer, **1**) or C-2 (2-isomer, **2**) of 5,8-dimethoxyl-1,4-naphthaquinone (DMNQ) scaffold (Fig. 1), showed promising potency in the inhibition of DNA topoisomerase-I. They displayed high reactivity in conjugation with glutathione, which was responsible for their cytotoxicity. Their inhibitory effects against L1210 cells were also demonstrated [2]. Interestingly, when a double bond contained in the side chain was incorporated to naphthaquinone core, its cytotoxicity to normal cells was reduced while its bioactivity kept unchanged [2]. Moreover, in combination with our previous report [8], 6-isomers were found to exhibit better anticancer activity than the corresponding 2-isomers. Unfortunately, researches on DMNQ with double bond contained in the side chain had been largely impeded, mainly lacking an efficient synthetic methodology to prepare such derivatives. Later on, we found that synthesis of 2-isomer of 5,8-*O*-dimethyl ether of shikonin was accessible through the direct methylation of shikonin [9], while its corresponding 6-isomer was formidable to be prepared. To acquire natural product shikonin with high optical purity, asymmetric synthesis and chiral resolution were proposed to prepare crucial intermediates, 5,8-*O*-dimethyl ether of shikonin derivatives, in our group [10, 11]. However, the reaction conditions of asymmetric synthesis were harsh and difficult to be controlled and its catalytic agents were so expensive. In the process of chiral resolution two enantiomers were too close to be separated and this operation was time-consuming. Based on the issues mentioned above, we took our efforts to develop an efficient synthetic approach to semi-synthesize an more excellent antitumor scaffold, 6-isomer of 5,8-dimethoxy1-1,4-naphthaquinones, bearing the synthetically challenging side chain such as 2-hydroxyl-5-methylpentenyl group (**13**).

**Scheme 1:**
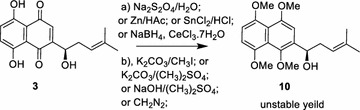
Direct synthesis of compound **10**

Modification of shikonin (**3**) was limited by its tendency to polymerize in the presence of acid, base, heat or temperature [2, 12–14]. Synthesis of compound **13** via direct methylation of shikonin failed as previously reported [2]. Selective preparation of compound **13** was ever pushed ahead when methoxymethyl was used as a protecting group, however, its application and scale were confined to deprotection and in situ oxidation. It was widely accepted that compound **13** could be synthesized in the form of mixture by oxidative demethylation of compound **10** [15]. Although 1,4,5,8-tetramethoxylnaphthaquinones could be obtained from 5,8-dihydroxyl-1,4-naphthoquinones using proper reducing agents and methylating ones [16], the presence of hydroxyl-containing side-chain on tetrahydroxylnaphthalene posed synthetically preparation of compound **10** a huge challenge [2, 17, 18] (Scheme 1). Therefore, to minimize its interference on the chemical behavior of the rest of the molecule, the side chain to be hidden was an appropriate approach to synthesize compound **10**. Previous researches on shikonin and its derivatives had demonstrated that cycloshikonin (**4**) was more stable than shikonin itself toward Lewis acid, strong base or high temperatures [19, 20]. The structure of cycloshikonin had been confirmed by Sankawa et al. [7] as 5,8-dihydroxyl-2-(5,6-dimethyl-2-tetrahydrofuranyl)-1,4-naphthoquinone. Although exposure to light, air or even high temperatures had little effect on racemization of shikonin as it existed in the solid form [21], little reports provided evidence for stability of chiral centre in the preparation for shikonin. Cyclization of the side chain of shikonin stood for a practical strategy for the preparation of compound **10**. We speculated that cycloshikonin would survive the reaction conditions where compound **4** could be converted into **5** while leaving *R*-configuration intact. In this paper, we described a targeting semi-synthesis of 6-isomers of 5, 8-*O*-dimethoxyl ether of shikonin via an ‘intra-molecular ring-closing/ring-opening’ strategy, coupled with introduction of a bulky substituent for regulating distribution of electron density on naphthoquinone scaffold. This methodology is being applied to explore and obtain a variety of more potential shikonin derivatives in search of promising candidate drugs for anticancer therapy.

## Results and discussion

**Scheme 2:**
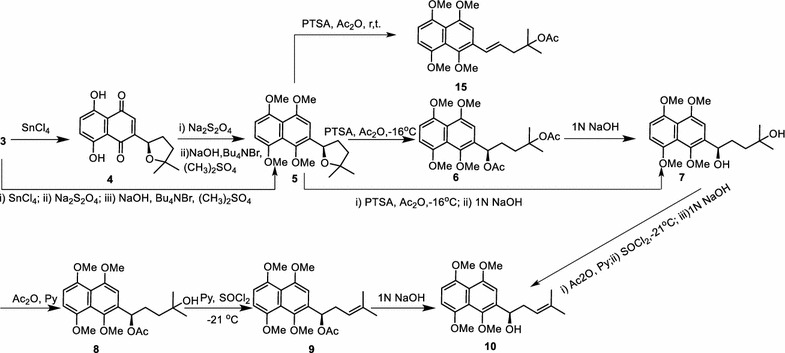
Synthesis of compound **10** via ring-closing/ring-opening strategy

A facile synthesis of 2-(1-hydroxyl-4-methyl-3-pentenyl)-1,4,5,8-tetramethoxynaphthalene (**10**) is illustrated in Scheme 2. Cyclization of the side chain of shikonin (**3**) to form cycloshikonin (**4**) had been well demonstrated by previous investigators [2, 22]. Cyclization of shikonin could proceed in the presence of *p*-toluensulfonic acid (PTSA) within 24 h, but the yield was low [22]. An alternative method that stannic chloride anhydrous was in place of PTSA gave compound **4** with the yield of 95% in 30 min. Noticeably, in the process of cyclization, shikonin with *R*-configuration didn’t change and e.e. value kept consistent, this was supported by the evidence that *S*-enantiomer of cycloshikonin analyzed with chiral HPLC didn’t appear (Additional file 1: Fig. S24).

Treatment of **4** with Na_2_S_2_O_4_ in a mixture of water and THF under N_2_ atmosphere provided the reduced cycloshikonin. Tetrabutylammonium bromide, NaOH and (CH_3_)_2_SO_4_ were subsequently added to a solution of the reduced cycloshikonin [17]. The ratio of NaOH to (CH_3_)_2_SO_4_ was found to be critical to the yield, and 4:1 was optimal. The above reaction mixture was stirred for 24 h under reflux to afford compound **5** with good repeatability in a more than 90% yield. Addition of tetrabutylammonium bromide, a phase transfer catalyst, was used to improve the solubility of the anion of the reduced shikonin, and then significantly increased the yield of compound **5**. However, a few alternative reductive methylation conditions failed to provide compound **5**. For instance, the most commonly used methylating agent CH_3_I in the presence of Ag_2_O failed to convert compound **4** to compound **5**. Reduced cycloshikonin was likely to be oxidized by Ag_2_O back to compound **4**, thus leading to the above observation. Treatment of reduced cycloshikonin with (CH_3_)_2_SO_4_ in the presence of K_2_CO_3_ and (CH_3_)_2_CO under various temperatures proved to be problematic as well. This could be due to reaction of cycloshikonin with (CH_3_)_2_CO to form 1,8-bridged or 4,5-bridged cycloshikonin, and then hampering further conversion [23]. Other reaction conditions including CH_2_N_2_, trimethylsilyldiazomethane (TMSCHN_2_) did not succeed in producing compound **5**, either.

Opening of furan ring of compound **5** was a crucial step, which was carried out with PTSA in Ac_2_O at low temperature to produce diacetyl **6** in an 88% yield. Higher temperature (> −16 °C) or room temperature resulted in yielding compound **15**, which is an isomer of compound **9** (Scheme 2). The amount of compound **15** increased with reaction temperature rising. Deprotection of acyl group from compound **6** by 1 N NaOH readily produced diol **7** with a yield of 99%. Subsequent acetylation of compound **7** with acetic anhydride in pyridine gave ester **8.** However, addition of 4-dimethylaminopyridine (DMAP) in this reaction gave rise to the undesired compound **6**. Compound **9** was produced from ester **8** in the presence of pyridine and thionyl chloride. Subsequently, treated with 1 N NaOH, compound **9** was hydrolyzed to compound **10** in a 94% yield. Since all the reaction conditions for synthesizing compound **10** were totally defined, several reactions were reasonably combined into one pot to spare reaction time and simplify purification operation. As demonstrated in Scheme 2, a concise synthetic route toward more efficient preparation of compound **10** was optimized from seven-step to three-step using “one-pot” strategy, the yield increased by 15%.

**Scheme 3:**
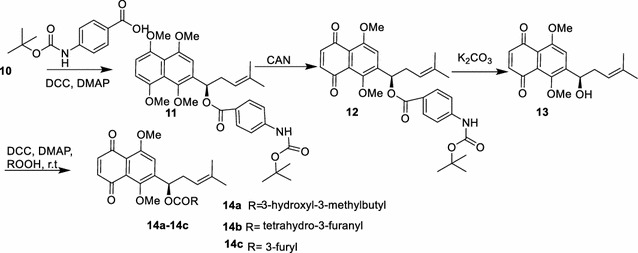
Regioselective synthesis of compound **13** and its derivatives **14a–14c**

As we known, oxidative demethylation of compound **10** in a solution of cerium(IV) ammonium nitrate (CAN) afforded the mixture of **13** and its positional isomer [2, 14]. In terms of the mechanism of CAN-mediated oxidative demethylation [24], introduction of a bulky substitute to 1-hydroxyl of the side chain to increase electron density of B ring contributed to its selective oxidation. Accordingly, esterification of compound **10** with a bulky group, 4-((tertbutoxycarbonyl)amino)benzoic acid in the presence of dicyclohexylcarbodiimide (DCC) and DMAP, gave rise to yield ester **11** in a 91% yield, which was selectively oxidative demethylated with CAN to compound **12**. The latter was hydrolyzed to target compound **13** in the presence of K_2_CO_3_ in a 92% yield. Finally, various 6-isomer ester derivatives (**14**) could be custom synthesized (Scheme 3). Three 6-isomer esters (**14a–14c**) [8] with very potent antitumor activities were taken as representative examples to demonstrate the advantageous application of the method (Scheme 3 and “Experimental Section”).

## Conclusions

In summary, we have developed selective semi-synthesis of 5,8-dimethoxyl-6-(1-hydroxyl-4-methylpentyl)-1,4-naphthaquinones (**13**) from natural product shikonin. The ring-closing/ring-opening strategy for obtaining the key intermediate, 2-(1-hydroxyl-4-methyl-3-pentenyl)-1,4,5,8- tetramethoxynaphthalene (**10**), was demonstrated to be effective, and the synthetic route was reasonably combined and optimized from seven-step to three-step. Cyclization of the side chain was applied to avoid the influence of hydroxyl-containing side-chain on reaction of its naphthaquinone core, and to ensure stereochemical retention of the configuration. A bulky-substituent-mediated oxidative demethylation was used to control the regioselective direction of 1,4,5,8-tetramethoxyshikonin derivatives. This work has provided a new targeting semi-synthetic route toward biologically important 6-isomer derivatives starting from shikonin.

## Experimental section

*General* Melting points (m.p.) were determined on a SGWX-4 micro-melting point apparatus and are uncorrected. NMR spectra were recorded on Varian Mercury-300 spectrometer (300 MHz for ^1^H and 75 MHz for ^13^C) or Varian Mercury-400 spectrometer (400 MHz for ^1^H and 100 MHz for ^13^C), chemical shifts of ^1^H and ^13^C spectra were recorded with tetramethylsilane as internal standard (CDC1_3_ δ_H_ 7.26, δ_C_ 77.2), and coupling constants were reported in hertz. Mass spectra were obtained on a ZAB-2F or JEOLDX-300 spectrometer. Optical rotations were measured on WZZ-3 polarimeter calibrated at the sodium D_line_ (598 nm). Reactions where exclusion of water was necessary were performed according to Ref. [25]. TLC was carried out on silica gel (GF254) under UV light. Column chromatography was run on silica gel (200–300 mesh) or alumina from Qingdao Ocean Chemical Factory.

### Shikonin (**3**)

Shikonin was extracted from *Lithospermum erythrorhizon* according to the procedure described by Birch [26]. Red-brownish needles, m.p. 145–146 °C (from CH_3_OH) (lit. m.p. 146–147 °C [27]); [α]_D_^25^ + 126.5° (c 0.2, C_6_H_6_), (lit. +138° [2]).

### (*R*)-5,8-dihydroxyl-2-(5,5-dimethyl-2-tetrahydrofuranyl)-1,4-naphthaquinone, (+) cycloshikonin (**4**)

Cycloshikonin was prepared from shikonin by the method proposed previously [2]. Yield: 98%. Solid, m.p. 78–80 °C (from CH_3_OH) (lit. m.p. 79–80 °C [2]); [α]_D_^25^ + 156.6° (c 0.33, CHCl_3_).^1^H NMR (300 MHz, CDCl_3_) *δ*: 12.53 (s, 1H, ArO*H*), 12.52 (s, 1H, ArO*H*), 7.23–7.19 (m, 3H, Ar*H*, Quinone*H*), 5.17 (dd, 1H, *J* = 6.3, 5.7 Hz, C*H*), 2.66–2.62 (m, 1H, C*H*_*2*_), 1.93–1.91 (m, 1H, C*H*_*2*_), 1.90–1.89 (m, 1H, C*H*_*2*_), 1.88–1.74 (m, 1H, C*H*_*2*_), 1.38 (s, 3H, C*H*_3_), 1.35 (s, 3H, C*H*_3_). ^13^C NMR (75 MHz, CDCl_3_) *δ*: 182.5, 181.5, 164.2, 163.7, 133.1, 132.0, 131.5, 131.4, 112.3, 111.9, 82.3, 74.7, 38.9, 33.7, 28.9, 28.0. MS (EI, *m/z*): 288 [M^+^], 255, 232, 219.

### (*R*)-2-(5,5-dimethyl-2-tetrahydrofuranyl)-1,4,5,8-tetramethoxynaphthalene (**5**)

To a solution of **4** (5 g, 17.3 mmol) and tetrabutylammonium bromide (1.0 g) in THF (160 mL) and water (80 mL) was added sodium dithionite (15.1 g, 86.3 mmol). After stirring for 15 min, NaOH (13.9 g, 0.35 mol) was added at room temperature. Dimethyl sulfate (21 mL) was added dropwise in 10 min, and the mixture was refluxing for 24 h. The product was separated by partitioning between water and DCM. The crude product was purified by column chromatography over silica gel with ethyl acetate/petroleum ether (1/4, v/v) to give 5.46 g of pale-yellow oil. Yield: 91%. [α] _D_^25^ +139.2^o^ (c 0.2, CHCl_3_); ^1^H NMR (300 MHz, CDCl3) *δ*: 7.12 (s, 1H, Ar*H*), 6.80 (s, 2H, Ar*H*), 5.52 (m, 1H, C*H*), 3.99 (s, 3H, OC*H*_3_), 3.95 (s, 3H, OC*H*_3_), 3.93 (s, 3H, OC*H*_3_), 3.75 (s, 3H, OC*H*_3_), 2.54–2.48 (m, 2H, C*H*_*2*_), 1.94–1.84 (m, 2H, C*H*_*2*_), 1.45 (s, 3H, C*H*_3_), 1.40 (s, 3H, C*H*_3_).^13^C NMR (75 MHz, CDCl_3_) *δ*: 152.7, 150.8, 149.6, 145.8, 133.2, 122.0, 119.5, 107.5, 106.9, 105.4, 80.4, 74.5, 61.7, 51.2, 56.3, 56.2, 38.5, 34.4, 28.3, 27.6. MS (ESI, %): 369 (M+Na^+^, 100), 401 (M^+^+NaOCH_3_, 45) and no parent peak was observed. HRMS (ESI) calcd. for C_20_H_27_O_5_^+^: 347.1853 [M+H]^+^; found: 347.1856.

### (*R*)-2-(1,4-diacetoxyl-4-methylpentyl)-1,4,5,8-tetramethoxynaphthalene (**6**) and 2-(4-acetoxyl-4-methyl-2-pentenyl)-1,4,5,8-tetramethoxynaphthalene (**15**)

A mixture of **5** (2 g, 5.8 mmo1) and *p*-toluenesulfonic acid monohydrate (1.14 g, 6 mmol) in acetic anhydride was allowed to stir overnight at −16 °C, and then the reaction mixture was diluted with methanol to quench excessive acetic anhydride and extracted with ethyl acetate. After the usual work-up, the residue was purified by column chromatography over silica gel with ethyl acetate/petroleum ether (1/3, v/v) as an eluent to give 2.28 g of pale-yellow oil. Yield: 88%. [α] _D_^25^ +142.2° (c 0.2, CHCl_3_). ^1^H NMR (300 MHz, CDCl_3_) *δ*: 6.85 (s, 1H, Ar*H*), 6.83 (s, 2H, Ar*H*), 6.32 (t, 1H, *J* = 7.8 Hz, C*H*), 3.94 (s, 3H, OC*H*_3_), 3.90 (s, 3H, OC*H*_3_), 3.88 (s, 3H, OC*H*_3_), 3.84 (s, 3H, OC*H*_3_), 2.12 (s, 3H, OCOC*H*_3_), 1.93–1.71 (m, 5H, C*H*_2_, OCOC*H*_3_), 1.41 (s, 3H, C*H*_3_), 1.39 (s, 3H, C*H*_3_). ^13^C NMR (75 MHz, CDCl_3_) *δ*: 170.5, 170.4, 153.8, 151.6, 150.7, 147.1, 130.9, 122.9, 121.1, 109.2, 108.1, 105.1, 81.9, 71.1, 62.7, 58.2, 57.7, 57.1, 37.1, 30.8, 26.2, 26.0, 22.6, 21.5. MS (ESI, %): 471 (M+Na^+^, 100), 503 (M^+^+NaOCH_3_, 31) and no parent peak was observed. HRMS (ESI) calcd. for C_24_H_33_O_8_^+^: 449.2170 [M+H]^+^, found: 449.2166.

The same operation as compound **6** was done at room temperature, major by-product **15** could be obtained as pale-yellow oil. ^1^H NMR (300 MHz, CDCl_3_) *δ*: 6.99 (s, 1H, Ar*H*), 6.90 (d, 1H, *J* = 15.6 Hz, C*H*=CH), 6.83 (s, 2H, Ar*H*), 6.28 (m, 1H, CH=C*H*), 4.00 (s, 3H, OC*H*_3_), 3.95 (s, 3H, OC*H*_3_), 3.84 (s, 3H, OC*H*_3_), 3.73 (s, 3H, OC*H*_3_), 2.78 (d, 2H, *J* = 6.6 Hz, C*H*_2_), 2.02 (s, 3H, OCOC*H*_3_), 1.52 (s, 6H, C*H*_3_). ^13^C NMR (75 MHz, CDCl_3_) *δ:* 171.2, 153.6, 151.3, 150.5, 147.2, 131.0, 122.2, 119.1, 109.5, 105.8, 105.3, 81.8, 71.0, 62.4, 58.0, 57.5, 57.3, 37.0, 30.6, 26.3, 26.1, 22.7. MS (ESI, %): 411 (M+Na^+^, 100), 443 (M^+^+NaOCH_3_, 38) and no parent peak was observed. HRMS (ESI) calcd. for C_22_H_29_O_6_^+^: 389.1959 [M+H]^+^, found: 389.1963.

### (*R*)-2-(1,4-dihydroxyl-4-methylpentyl)-1,4,5,8-tetramethoxynaphthalene (**7**)

Hydrolysis of **6** (1.5 g, 3.4 mmol) in 1 N sodium hydroxide (160 mL) and methanol (50 mL) was stirred at 0–5 °C for 12 h under a nitrogen atmosphere. Ethyl acetate was added to dilute the reactive mixture. Organic layer was washed with 4% HCl, water and saturated brine respectively, dried over anhydrous MgSO_4_ and evaporated to give the crude product, which was purified by column chromatography with ethyl acetate/petroleum ether (1/2, v/v) to produce 1.23 g of pale-yellow oil. Yield: 99%. [α] _D_^25^ + 143.7° (c 0.2, CHCl_3_). ^1^H NMR (300 MHz, CDCl_3_) δ: 7.02 (s, 1H, Ar*H*), 6.81 (s, 2H, Ar*H*), 5.24 (dd, 1H, *J* = 5.4, 5.1 Hz, C*H*), 3.92 (s, 9H, OC*H*_3_), 3.72 (s, 3H, OC*H*_3_), 1.95–1.54 (m, 4H, C*H*_2_), 1.22 (s, 6H, C*H*_3_). ^13^C NMR (75 MHz, CDCl_3_) δ: 152.4, 150.4, 149.2, 145.3, 133.4, 121.5, 119.2, 107.4, 106.7, 105.0, 69.5, 68.0, 61.7, 56.8, 56.1, 55.8, 39.1, 32.1, 28.7, 28.0. MS (ESI, %): 387 (M+Na^+^, 100), 419 (M^+^+NaOCH_3_, 25), 751 (2M+Na^+^, 38) and no parent peak was observed. HRMS (ESI) calcd. for C_20_H_29_O_6_^+^: 365.1959 [M+H]^+^, found: 365.1956.

### (*R*)-2-(1-acetoxyl-4-hydroxyl-4-methylpentyl)-1,4,5,8-tetramethoxynaphthalene (**8**)

Acetic anhydride (10 mL) was added to a solution of **7** (1.20 g, 3.3 mmol) dissolved in pyridine (20 mL) at 0–5 °C, and the mixture was stirred for 2 h at the same temperature. Excess of the reagents were removed by HCl, NaHCO_3_, water and saturated brine in order, and then the crude product was purified by column chromatography with ethyl acetate/petroleum ether (1/1, v/v) to give 1.28 g of yellowish oil. Yield: 95%. [α] _D_^25^ + 145.7° (c 0.1, CHCl_3_). ^1^H NMR (300 MHz, CDCl_3_) *δ:* 6.86 (s, 1H, Ar*H*), 6.83 (s, 2H, Ar*H*), 6.36 (dd, 1H, *J* = 5.7, 6.0 Hz, C*H*), 3.93 (s, 6H, OC*H*_3_), 3.88 (s, 3H, OC*H*_3_), 3.83 (s, 3H, OC*H*_3_), 2.11 (s, 3H, OCOC*H*_3_), 2.04–1.25 (m, 4H, C*H*_2_), 1.18 (s, 3H, C*H*_3_), 1.17 (s, 3H, C*H*_3_). ^13^C NMR (75 MHz, CDCl_3_) *δ*: 170.5, 153.7, 151.7, 150.4, 146.6, 131.1, 120.7, 120.6, 109.0, 107.9, 105.8, 71.3, 70.8, 62.7, 58.2, 57.6, 57.1, 39.6, 31.3, 29.9, 29.3, 21.6. MS (ESI, %): 429 (M+Na^+^, 100), 461 (M^+^+NaOCH_3_, 15) and no parent peak was observed. HRMS (ESI) calcd. for C_22_H_31_O_7_^+^: 407.2064 [M+H]^+^, found: 407.2067.

### (*R*)-2-(1-acetoxyl-4-methyl-3-pentenyl)-1,4,5,8-tetramethoxynaphthalene (**9**)

Compound **8** (1.20 g, 2.96 mmol) in dry pyridine (50 mL) was cooled to −21 °C with ice-salted water, subsequently thionyl chloride was added. The reaction mixture was stirred at −21 °C for 15 min, and then poured into ice-water. The mixture was extracted with ethyl acetate twice, and organic layer combined was washed with water, saturated brine, and dried over anhydrous Na_2_SO_4_ and concentrated under reduced pressure. Column chromatography of the residue over alumina with ethyl acetate/petroleum ether (1/3, v/v) gave 945.8 mg of pale-yellow oil. Yield: 82.2%. [α] _D_^25^ +124.5 (c 0.2, CHCl_3_). ^1^H NMR (300 MHz, CDCl_3_) *δ:* 6.87 (s, 1H, Ar*H*), 6.82 (s, 2H, Ar*H*), 6.34 (dd, 1H, *J* = 4.5, 6.0 Hz, C*H*), 6.15 (t, 1H, *J* = 4.5 Hz, C*H*), 3.93 (s, 6H, OC*H*_3_), 3.86 (s, 3H, OC*H*_3_), 3.83 (s, 3H, OC*H*_3_), 2.59–2.54 (m, 2H, C*H*_2_), 2.10 (s, 3H, OCOC*H*_3_), 1.65 (s, 3H, C*H*_3_), 1.55 (s, 3H, C*H*_3_). ^13^C NMR (75 MHz, CDCl3) *δ:* 170.4, 153.5, 151.6, 150.8, 147.1, 134.8, 130.9, 122.9, 120.9, 119.4, 109.0, 108.2, 105.6, 71.1, 62.7, 58.1, 57.7, 57.3, 34.8, 25.9, 21.5, 18.1. MS (ESI, %): 411 (M+Na^+^, 100), 443 (M^+^+NaOCH_3_, 18) and no parent peak was observed. HRMS (ESI) calcd. for C_22_H_28_O_6_Na^+^: 411.1778 [M+Na]^+^, found: 411.1776.

### (*R*)-2-(1-hydroxyl-4-methyl-3-pentenyl)-1,4,5,8-tetramethoxynaphthalene (**10**)

Hydrolysis of **9** (1 g, 2.6 mmol) in 1 N sodium hydroxide (100 mL) and methanol (50 mL) was stirred at 0–5 °C for 12 h under a nitrogen atmosphere. Ethyl acetate was added to dilute the reactive mixture. Organic layer was washed with water and saturated brine, and dried over anhydrous MgSO4, and then evaporated under reduced pressure. The crude product was purified by column chromatography over silica gel with ethyl acetate/petroleum ether (1/4, v/v) to obtain 839.2 mg of desirable compound. Yield: 94%. [α]_D_^25^ +149.2° (c 0.24, CHCl_3_). ^1^H NMR (300 MHz, CDCl_3_) *δ:* 7.02 (s, 1H, Ar*H*), 6.82 (s, 2H, Ar*H*), 5.33 (m, 2H, C*H*, C*H*), 3.95 (s, 6H, OC*H*_3_), 3.93 (s, 3H, OC*H*_3_), 3.76 (s, 3H, OC*H*_3_), 2.55–2.51 (m, 2H, C*H*_2_), 1.72 (s, 3H, C*H*_3_), 1.65 (s, 3H, C*H*_3_). ^13^C NMR (75 MHz, CDCl_3_) *δ:* 153.6, 151.7, 150.5, 146.8, 135.4, 134.2, 122.9, 120.5, 108.6, 108.1, 106.4, 68.8, 63.0, 58.6, 58.1, 57.4, 57.2, 37.4, 25.1, 18.2. MS (ESI, %): 369 (M+Na^+^, 100), 401 (M^+^+NaOCH_3_, 38) and no parent peak was observed. HRMS (ESI) calcd. for C_20_H_27_O_5_^+^: 347.1853 [M+H]^+^, found: 347.1856.

### (*R*)-4-methyl-1-(1,4,5,8-tetramethoxynaphthalen-2-yl)pent-3-en-1-yl-4-((tertbutoxycarbonyl)amino) benzoate (**11**)

To a stirred solution of **10** (2.0 g, 5.8 mmol) and 4-((tertbutoxycarbonyl)amino)benzoic acid (1.66 g, 7.0 mmol) in anhydrous DCM were added DCC (1.4 g, 7.0 mmol) and DMAP (350 mg, 2.9 mmol). After stirring overnight at room temperature, petroleum ether was added into the reaction mixture to facilitate precipitates at 4 °C, and then the solution was filtered, and concentrated in vacuo. The residue was purified by flash chromatography to afford 2.99 g of **11** as colorless oil. Yield: 91%. [α] ^D25^ +139.7° (c 0.25, CHCl_3_). ^1^H NMR (400 MHz, CDCl3) *δ:* 7.93 (d, 2H, *J* = 0.8 Hz, Ar*H*), 7.37 (d, 2H, *J* = 0.8 Hz, Ar*H*), 6.86 (s, 1H, Ar*H*), 6.73 (s, 2H, Ar*H*), 6.42–6.47 (m, 1H, C*H*), 5.14 (t, *J* = 7.2 Hz, 1H, C*H*), 3.85 (s, 3H, OC*H*_3_), 3.82 (s, 3H, OC*H*_3_), 3.78 (s, 6H, OC*H*_3_), 2.55–2.67 (m, 2H, C*H*_2_), 1.56 (s, 3H, C*H*_3_), 1.49 (s, 3H, C*H*_3_), 1.42 (s, 9H, C*H*_3_). ^13^C NMR (100 MHz, CDCl_3_) *δ:* 164.6, 152.1, 151.0, 150.4, 149.4, 145.5, 141.6, 133.5, 130.6, 129.7, 123.6, 121.5, 119.3, 118.1, 116.3, 107.4, 106.4, 104.8, 80.0, 70.3, 61.4, 56.7, 56.1, 55.9, 33.6, 27.1, 24.7, 17.0. HRMS (ESI), calcd. for C_32_H_40_NO_8_^+^: 566.2748 [M+H]^+^, found: 566.2744.

### (*R*)-6-(1-(4-(*N*-(tertbutoxycarbonyl)amino)benzoyloxy)-4-methylpent-3-en-1-yl)-5,8-dimethoxy-1,4-naphthoquinone (**12**)

A solution of CAN (3.69 g, 6.8 mmol) in water (20 mL) was added dropwise to a stirred solution of **11** (3.28 g, 5.8 mmol) in the ice bath. The mixture was risen up to room temperature, and stirred for additional 10 min, and then diluted with water and ethyl acetate. Organic layer was separated and aqueous layer was extracted with ethyl acetate (2 × 100 mL). The combined organic extracts were washed with saturated brine (150 mL), and dried over anhydrous Na_2_SO_4_, and then concentrated under reduced pressure. The residue was purified by column chromatography with ethyl acetate/petroleum ether (1/1, v/v) to give 3.1 g of compound **12** as yellow oil. Yield: 91%, ^1^H NMR (400 MHz, CDCl_3_) *δ:* 7.94 (d, *J* = 0.8 Hz, 2H, Ar*H*), 7.42 (d, *J* = 0.8 Hz, 2H, Ar*H*), 7.23 (s, 1H, Ar*H*), 6.70 (s, 2H, Quinone*H*), 6.22 (t, *J* = 4.0 Hz, 1H, C*H*), 5.14 (t, *J* = 6.8 Hz, 1H, C*H*), 3.91 (s, 3H, OC*H*_3_), 3.80 (s, 3H, OC*H*_3_), 2.59–2.64 (m, 1H, C*H*_*2*_), 2.49–2.56 (m, 1H, C*H*_*2*_), 1.61 (s, 3H, C*H*_3_), 1.50 (s, 3H, C*H*_3_), 1.44 (s, 9H, C*H*_3_). ^13^C NMR (100 MHz, CDCl_3_) *δ:* 184.8, 184.3, 165.3, 156.1, 152.2, 150.6, 144.9, 143.2, 138.9, 137.8, 135.8, 130.8, 125.2, 123.9, 120.1, 118.2, 117.5, 116.6, 81.3, 71.2, 62.0, 56.6, 34.1, 28.2, 25.8, 17.9. HRMS (ESI) calcd. for C_30_H_34_NO_8_^+^: 536.2279 [M+H]^+^, found: 536.2284.

### (*R*)-5,8-dimethoxyl-6-(1-hydroxyl-4-methylpentyl)-1,4-naphthaquinones (**13**)

A solution of K_2_CO_3_ (6.6 g, 48.0 mmol) was added dropwise to a stirred solution of **12** (12.9 g, 24.0 mmol) dissolved in THF (250 mL) at ice-bath. The reaction mixture was stirred for 2 h at the same temperature. The progression was monitored by TLC. After completion, the mixture was neutralized with statured NH_4_Cl solution, and then diluted with water and ethyl acetate. Organic layer was separated and aqueous layer was extracted with ethyl acetate (2 × 150 mL). The combined organic extracts were washed with saturated brine (200 mL), dried over anhydrous Na_2_SO_4_ and concentrated under reduced pressure. The residue was purified by column chromatography with ethyl acetate/petroleum ether (1/1, v/v) as an eluent to give 6.98 g of yellowish oil **13**. Yield: 92%. [α] _D_^25^ +48.5° (c 0.5, CHCl_3_). ^1^H NMR (300 MHz, CDCl_3_) *δ:* 7.55 (s, 1H, Ar*H*), 6.79 (d, 2H, *J* = 3.0 Hz, Quinone*H*), 5.24 (t, 1H, *J* = 6.0 Hz, C*H*), 5.10 (t, 1H, *J* = 3.0 Hz, C*H*), 3.97 (s, 3H, OC*H*_3_), 3.89 (s, 3H, OC*H*_3_), 2.35–2.19 (m, 2H, C*H*_2_), 1.76 (s, 3H, C*H*_3_), 1.65 (s, 3H, C*H*_3_). ^13^C NMR (75 MHz, CDCl_3_) *δ:* 185.1, 184.5, 156.5, 150.9, 147.9, 139.2, 137.9, 136.9, 125.1, 68.8, 62.4, 56.9, 37.2, 26.1, 18.2. MS (ESI,  %): 317 (M+H^+^, 12.5), 339 (M+Na^+^, 30), 371 (M^+^+NaOCH_3_, 100). HRMS (ESI) calcd. for C_18_H_20_O_5_Na^+^: 339.1203 [M+Na]^+^, found: 339.1207.

### (*R*)-1-(1,4-dimethoxy-5,8-dioxo-5,8-dihydronaphthalen-2-yl)-4-methylpent-3-en-1-yl 3-hydroxy-3-methylbutanoate (**14a**)

To a stirred solution of **13** (3.16 g, 10.0 mmol) and 3-hydroxy-3-methylbutanoic acid (1.30 g, 11.0 mmol) in anhydrous DCM were added DCC (2.27 g, 11.0 mmol) and DMAP (350 mg, 2.9 mmol). TLC was applied to monitor the progression. After completion, petroleum ether was added into the reaction mixture to facilitate precipitates at 4 °C, and filtered to remove the insoluble substance, and concentrated in vacuo. The residue was purified by flash chromatography to afford 2.54 g of **14a** as yellow oil. Yield: 61%. [α] _D_^25^ +59.3° (c 0.4, CHCl_3_). ^1^H NMR (300 MHz, CDCl_3_) *δ*: 7.27 (s, 1H, Ar*H*), 6.67 (d, 2H, *J* = 3.0 Hz, Quinone*H*), 6.18 (m, H, C*H*), 5.04 (t, 1H, *J* = 8.1 Hz, C*H*), 3.95 (s, 3H, OC*H*_3_), 3.94 (s, 3H, OC*H*_3_), 2.58–2.38 (m, 4H, 2 × C*H*_2_), 1.68 (s, 3H, C*H*_3_), 1.55 (s, 3H, C*H*_3_), 1.29 (s, 3H, C*H*_3_), 1.26 (s, 3H, C*H*_3_). ^13^C NMR (75 MHz, CDCl_3_) *δ:* 187.6, 186.5, 173.2, 152.1, 138.7, 134.2, 132.0, 124.1, 119.7, 115.2, 114.3, 70.9, 70.0, 62.3, 55.4, 42.1, 32.4, 29.2, 24.4, 18.1. HRMS (ESI): calcd for C_23_H_29_O_7_^+^: 417.1908 [M+H]^+^, found: 417.1902. These data were in accordance with the literature [8].

### (*R*)-1-(1,4-dimethoxy-5,8-dioxo-5,8-dihydronaphthalen-2-yl)-4-methylpent-3-en-1-yl tetrahydrofuran-3-carboxylate (**14b**)

The preparation procedure for compound **14b** was similar to that of compound **14a**, and tetrahydrofuran-3-carboxylic acid was substituted for 3-hydroxy-3-methylbutanoic acid. Yield: 71%. [α] _D_^25^ +56.3° (c 0.5, CHCl_3_). ^1^H NMR (300 MHz, CDCl_3_) *δ:* 7.24 (d 1H, *J* = 3.0 Hz, Ar*H*), 6.78 (d, 2H, *J* = 3.3 Hz, Quinone*H*), 6.16 (m, 1H, C*H*), 5.11 (t, 1H, *J* = 6.3 Hz, C*H*), 4.02–3.79 (m, 10H, 2 × OC*H*_3_, 2 × OC*H*_2_), 3.19 (m, 1H, C*H*), 2.53–2.44 (m, 2H, C*H*_2_), 1.68 (s, 3H, C*H*_3_), 1.54 (s, 3H, C*H*_3_). ^13^C NMR (75 MHz, CDCl_3_) *δ:* 186.3, 186.2, 173.2, 152.3, 152.2, 138.7, 134.1, 132.2, 120.1, 119.8, 114.3, 114.2, 75.6, 70.9, 70.5, 61.8, 55.4, 42.5, 32.3, 31.6, 24.5, 18.3. HRMS (ESI) calcd for C_23_H_27_O_7_^+^: 415.1751 [M+H]^+^; found: 415.1756. These data were in accordance with the literature [8].

### (*R*)-1-(1,4-dimethoxy-5,8-dioxo-5,8-dihydronaphthalen-2-yl)-4-methylpent-3-en-1-yl furan-3-carboxylate (**14c**)

The preparation procedure of compound **14c** was similar to that of compound **14a**, 3-hydroxy-3-methylbutanoic acid was replaced with furan-3-carboxylic acid. Yield: 55%. [α] _D_^25^ +48.3° (c 0.3, CHCl_3_). ^1^H NMR (300 MHz, CDCl_3_) *δ:* 8.10 (d, 1H, *J* = 1.2 Hz, Furanyl*H),* 7.49 (d, 1H, *J* = 1.2 Hz, Furanyl*H)*, 7.29 (s, 1H, Ar*H*), 6.82 (d, 2H, *J* = 3.0 Hz, Quinone*H*), 6.80 (s, 1H, Furanyl*H*), 6.52 (dd, 1H, *J* = 4.8, 4.8 Hz, C*H*), 5.19 (t, 1H, *J* = 7.5 Hz, C*H*), 3.97 (s, 3H, OC*H*_3_), 3.94 (s, 3H, OC*H*_3_), 2.63–2.57 (m, 2H, C*H*_2_), 1.69 (s, 3H, C*H*_3_), 1.58 (s, 3H, C*H*_3_). ^13^C NMR (75 MHz, CDCl_3_) *δ:* 187.1, 186.9, 159.2, 152.1, 148.6, 143.9, 137.6, 137.5, 134.2, 132.1, 119.7, 119.6, 118.3, 114.9, 114.4, 110.6, 70.1, 62.4, 55.8, 32.2, 24.4, 18.3. HRMS (ESI): calcd. for C_23_H_23_O_7_^+^: 411.1438 [M+H]^+^, found: 411.1442. These data were in accordance with the literature [8].

### Chiral HPLC analysis conditions for shikonin and its derivatives

The chiral HPLC column applied (150 × 4.6 mm) was Sino-Chiral OD [No. 0A02014-C (Packing cellulose-tris (3,5-dimethylphenyl carbamate)], which was purchased from FunSea Beijing Technology Co. Ltd (Beijing). All the separations were performed at ambient temperature. The mobile phase, hexane–isopropanol (80:20, v/v) was degassed before application. To obtain sufficient resolution of shikonin, alkannin and their derivatives, the flow rate of mobile phase was adjusted to 0.65 mL/min and injection volume was set at 5 μL.

## Additional file

**Additional file 1.** Additional figures.
