# Supplementary material for: Regioselective semi-synthesis of 6-isomers of 5,8-O-dimethyl ether of shikonin derivatives via an ‘intramolecular ring-closing/ring-opening’ strategy as potent anticancer agents
Source: Chem Cent J. 2017 Aug 2;11:74. doi: 10.1186/s13065-017-0306-0 (PMC5540739; doi:10.1186/s13065-017-0306-0)
Supplement: Supplementary file 1 — Additional file 1. Additional figures. [file 13065_2017_306_MOESM1_ESM.docx]

**Regioselective semi-synthesis of 6-isomers of 5, 8-*O*-dimethyl ether of shikonin derivatives *via* an 'intramolecular ring-closing/ ring-opening' strategy as potent anticancer agents**

# Li Zhou,^a^ Xu Zhang^2^ andWen Zhou^3^*

1College of Science, Hunan Agricultural University, Furong, Changsha, Hunan Province, China, 410128

2College of Forestry and landscape Architecture, South China Agricultural University, 483, Wushan Rd, Guangzhou, Guangdong province, China, 510642.

3 School of Chinese MeteriaMedica, Guangzhou University of Chinese Medicine, E. 232, University town, Waihuan Rd, Panyu, Guangzhou, Guangdong province, China, 510006

*E-mail:[zhouwen60@126.com](mailto:zhouwen60@126.com)

**Table of contents**

[^1^H and ^13^C NMR spectra for compounds 3~13 2-](#_Toc463638168)11

**^1^H NMR spectra for compounds 14 a~14c …………………………………………………………..12-13**

**Chiral HPLC of shikonin and Cycloshikonin…………………………………………………14**

**MS (ESI) spectra for compounds 7~9, 13………………………………………………………14-16**

**Fig. S1** ^1^H NMR Spectra for compound **3**

**
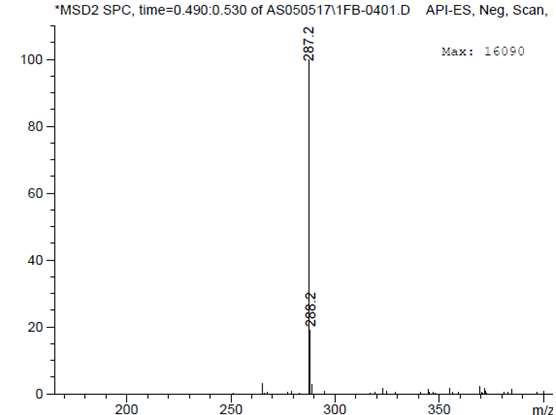
**

**Fig. S2** MS Spectra for compound **3**

**Fig. S3** ^1^H NMR Spectra for compound **4**

**Fig. S4** ^1^H NMR Spectra for compound **5**

**Fig. S5** ^13^C NMR Spectra for compound **5**

**Fig. S5** ^1^H NMR Spectra for compound **6**

**Fig. S6** ^13^C NMR Spectra for compound **6**

**Fig. S7** ^1^H NMR Spectra for compound **15**

**Fig. S8** ^1^H NMR Spectra for compound **7**

**Fig. S9** ^13^C NMR Spectra for compound **7**

**Fig. S10** ^1^H NMR Spectra for compound **8**

**Fig. S11** ^13^C NMR Spectra for compound **8**

**Fig. S12** ^1^H NMR Spectra for compound **9**

**Fig. S13** ^13^C NMR spectra for compound **9**

**Fig. S14** ^1^H NMR spectra for compound **10**


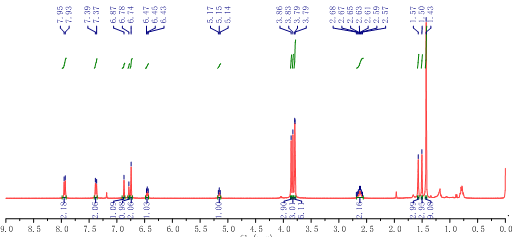


**Fig. S15** ^1^H NMR Spectra for compound **11**


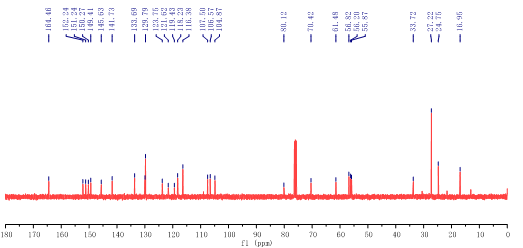


**Fig. S16** ^13^C NMR Spectra for compound **11**


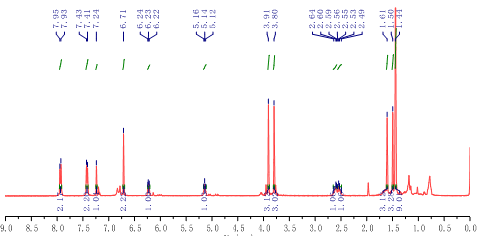


**Fig. S17** ^1^H NMR Spectra for compound **12**


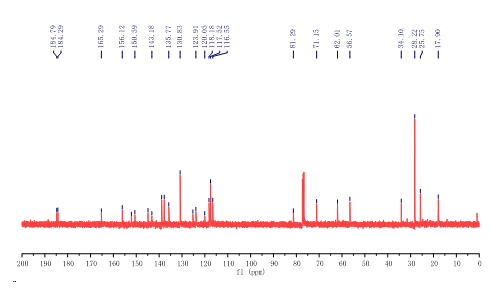


**Fig. S18** ^13^C NMR Spectra for compound **12**


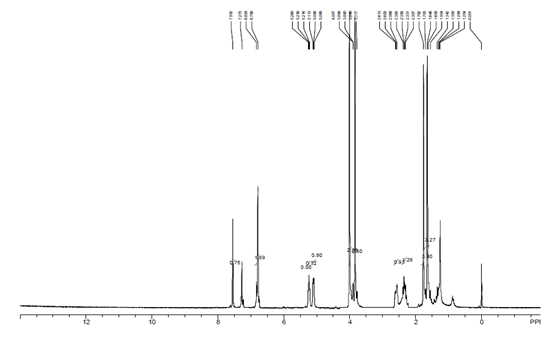


**Fig. S19** ^1^H NMR Spectra for compound **13**


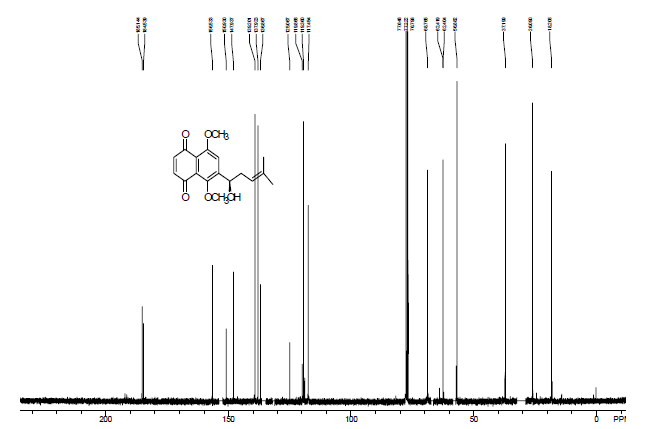


**Fig. S20** ^13^C NMR Spectra for compound **13**

**Fig. S21** ^1^H NMR Spectra for compound **14a**

**Fig. S22** ^1^H NMR Spectra for compound **14b**

**Fig. S23** ^1^H NMR Spectra for compound **14c**


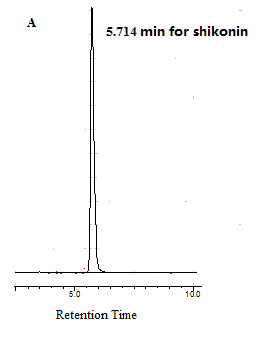

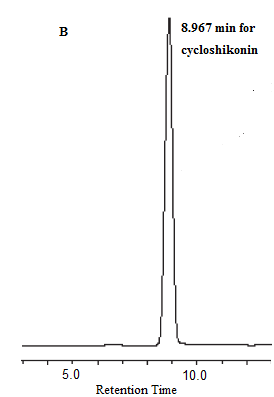


**Fig. S24.** Shikonin and cycloshikonin analysized by chiral HPLC

(A for shikonin and B for cycloshikonin)

M^+^+NaOCH_3_

**Fig. S 25** MS (ESI) Spectra for compound **7**

M^+^+NaOCH_3_

**Fig. S 26**  MS (ESI) Spectra for compound **8**

M^+^+NaOCH_3_

**Fig. S 27**  MS (ESI) Spectra for compound **9**

M^+^+NaOCH_3_

**Fig. S 28**  MS (ESI) Spectra for compound **13**
